# What can we learn from geographical comparisons of childhood cancer survival?

**DOI:** 10.1038/sj.bjc.6603749

**Published:** 2007-04-17

**Authors:** K Pritchard-Jones, C Stiller

**Affiliations:** 1 Section of Paediatric Oncology, Institute of Cancer Research & Royal Marsden NHS Foundation Trust, Downs Road, Sutton, Surrey, SM2 5PT, UK; 2 Childhood Cancer Research Group, Department of Paediatrics, University of Oxford, 57 Woodstock Rd, Oxford, OX2 6HJ, UK

**Keywords:** population based, cancer registries, paediatric cancer, international comparisons

## Abstract

With improvements in treatment for childhood cancer, comparisons of survival rates between countries have become important to inform future health policies and treatment strategies. Population-based cancer registry data are viewed as the gold standard for such comparisons, but even these have potential confounding factors. Here, we review the interpretation of recent geographical comparisons of childhood cancer survival from the viewpoint of the British Isles, a region with a 45-year record of national population-based cancer registration and a national childhood cancer clinical trials organisation in place for nearly 30 years. Using national data on referral patterns to tertiary paediatric oncology centres, we explore some of the reasons for lower survival rates in the past for some tumour groups and anticipate continued improvement in the next decade. Participation in international clinical trials coincided with rapid gains in survival for hepatoblastoma. This exemplifies the potential benefits of international collaborative clinical research, particularly for rare subgroups.

Successful treatment of childhood cancer relies on many factors, some of which are inherent to each tumour's biology but some of which can be more readily influenced, such as early recognition of concerning symptoms by families and physicians, referral practices and the availability and implementation of optimal, usually standardised, treatment protocols. Survival from childhood cancer in the UK has been the subject of international comparisons since the EUROCARE studies in the 1990s (Coebergh *et al*, 2001; Terracini *et al*, 2001). Most recently, interregional comparisons of both incidence and survival across Europe have been made using the Automated Childhood Cancer Information System (ACCIS) (Steliarova-Foucher *et al*, 2004). In this study, the regions were defined largely according to UN definitions. As the UK and Republic of Ireland have national population-based cancer registration and contributed large numbers of cases, their data were analysed and presented as a single group under the heading of ‘British Isles’ (BI), separately from the North European region (Pritchard-Jones *et al*, 2006). This allows comparisons of the basic demographics and outcome for childhood cancer treatment in the BI to be made with other European regions. Although such comparisons may be useful in assessing the effectiveness of cancer services for children, they also raise questions for those subgroups where outcomes are significantly different from the European average. We consider here possible reasons for these differences, particularly whether they are more likely to be attributable to differences in disease occurrence or patterns of care or to be artefacts arising from variations in cancer registry practice across Europe. In the ACCIS analyses, the most recent year of diagnosis for most of the BI was 1995 (Steliarova-Foucher *et al*, 2006), but the results are also discussed in the context of trends in survival in Great Britain up to 2000 and rates of referral to paediatric oncology centres up to 2002.

Before drawing any conclusions from these analyses, one must take into consideration the comparability of the data sources. Comparability issues were considered carefully in deciding which registry data should be included in the ACCIS analyses. Cancer registration is a complex process that relies on comprehensive access to hospital and population records, however, and comparability is not yet perfect owing to national variations in registration practices and access to personal data (Pritchard-Jones *et al*, 2006; Steliarova-Foucher *et al*, 2006). For example, incidence and survival figures in registries without access to national mortality databases (as in Germany, France, Netherlands, Italy and Spain) may overestimate survival owing to incomplete follow-up for vital status (Steliarova-Foucher *et al*, 2006). The data for the BI suffer less from this, as legislation permits linkage to databases of identifiable deceased individuals. The ACCIS analyses refer to all diagnoses in the International Classification of Childhood Cancer (Kramarova and Stiller, 1996), that is, all malignant neoplasms and most types of non-malignant intracranial and intraspinal tumours. Although the latter are collected routinely by most cancer registries, there are some variations between registries and hence also between geographical regions, whose implications for interpretation of the results are discussed below.

In the most recent period of the ACCIS analysis (1988–1997), observed overall 5-year survival for 49 651 children aged under 15 years grouped into the five European regions was 72% (Sankila *et al*, 2006). Observed survival was used in place of relative survival, as competing causes of death are rare in children in Western populations and relative survival would exceed observed survival by less than one percentage point. For comparison, the 5-year relative survival was 75% in the USA for patients diagnosed in 1985–1999 (Ries *et al*, 2003). Observed survival ranged from 77% in the North, through 75% in the West, 72% in the South, 71% in the BI to 62% in the East (Sankila *et al*, 2006). The survival curves tested by log rank were significantly different for the BI compared individually with North, West or East, but were not distinguishable from survival in the South. For this analysis, the regions included data from the following countries: North (Denmark, Iceland, Finland, Norway), West (France, Germany (East and West 1991–1997; former West Germany only 1988–1990), Netherlands, Switzerland), South (Italy, Malta, Slovenia, Spain), BI (Ireland, England, Northern Ireland, Scotland, Wales) and East (Belarus, Estonia, Hungary, Slovakia). Trends in survival were analysed over the 20-year period 1978–1997 with some slight differences in the regional data sets: BI (England, Scotland and Wales), East (Estonia, Hungary, Slovakia, former East Germany, 1978–1987), South (Italy, Slovenia, Spain) (Magnani *et al*, 2006) (Figure 1). The relative ranking of regions did not alter over this longer study period. Highly significant increases in observed survival were seen in all European regions, with the most rapid rise in the East. For all neoplasms, the BI had a 5-year survival of 74% in the most recent period, 1993–97 (Magnani *et al*, 2006). Survival has continued to increase, reaching 77% in Great Britain (which accounts for about 90% of cases in the BI) during 1996–2000 (Figure 2) (Stiller, 2007).

The reported survival differences between the BI and some other European regions are small in absolute terms. Some of this variation may be artefactual due to several possible factors. First, it should be noted that the BI had the lowest incidence rates in Europe for all childhood cancers combined. The age-standardised rate was 131.1 per million compared with 138.5 per million for Europe as a whole with the highest rate of 160.1 per million in the North (Stiller *et al*, 2006a). The deficit was found among boys and girls at all ages throughout childhood. It was most marked in the first year of life, with more than half of the difference from the European average being accounted for by the relatively low incidence of neuroblastoma among infants (Stiller *et al*, 2006a). Incidence rates may influence survival in several ways. For example, survival will increase if there is ‘overdiagnosis’ of cases with a very favourable prognosis that may not otherwise have presented clinically, as has been observed for neuroblastoma (Spix *et al*, 2006). Variations in diagnostic and registration practices for brain tumours may contribute to higher survival in those regions covered by registries with a higher total incidence resulting from inclusion of a higher proportion of non-malignant cases (Peris-Bonet *et al*, 2006).

The significance of comparisons of survival among North, South, West and BI for the 12 main groups and the principal subgroups of childhood cancers is weakened by their ‘*post hoc*’ nature and the fact that the large number of comparisons means that some significant results would be expected to arise by chance. In general, the highest survival figures were often observed in the North. Differences were seen between the BI and the region(s) with highest 5-year survival for sympathetic nervous system tumours, renal tumours and soft tissue sarcomas (Pastore *et al*, 2006a, 2006b; Spix *et al*, 2006). Differences in observed survival were also noted for the following subgroups: neuroblastoma, Wilms tumour, acute lymphoblastic leukaemia (ALL), osteosarcoma, primitive neuroectodermal tumours/medulloblastoma and ‘glioma-related’ brain tumours, although the composition of this last subgroup is too heterogeneous for meaningful comparison, as explained below (Coebergh *et al*, 2006; Peris-Bonet *et al*, 2006; Pastore *et al*, 2006a; Spix *et al*, 2006; Stiller *et al*, 2006b). Despite these limitations, possible reasons for these potential differences in certain tumour groups merit further consideration, as they may be informative in stimulating assessment of factors with the potential to influence effectiveness of care.

For neuroblastoma, it has been recognised previously that the BI has a lower total incidence that includes a relatively high proportion of older children with disseminated disease, compared with some other Western European countries (Powell *et al*, 1998). This pattern of presentation may be partially explained by the influence of screening programmes and differences in the use of diagnostic ultrasound in paediatric primary care in other European countries and may, to some extent, explain the lower survival in Great Britain during 1988–1995. Survival has improved consistently since this period (Figure 3). This improvement is not easily explained as the same clinical trial for the major subgroup of children with stage 4 disease (ENSG V, 1990–1999) ran throughout both periods. Further analysis would require data that are beyond the current scope of cancer registries, such as participation rates in randomised clinical trials, where the more intensive experimental arm subsequently showed a survival benefit.

Survival for children with renal tumours in the BI, comprising mainly Wilms tumours, remained static in the BI during the ACCIS study period and was similar to rates in the South, but inferior to rates in the North and West (Pastore *et al*, 2006a). This overall picture suggesting no change is confounded by an unexplained fall in survival in the early 1990s compared with the late 1980s (Stiller, 2007). Overall survival has subsequently improved markedly, which may be partially attributable to the introduction of a national strategy for treatment of relapsed Wilms tumour in the late 1990s (Figure 3).

Geographical comparisons of survival from soft tissue sarcomas are complicated by the fact that this diagnostic group encompasses a diverse collection of histological entities with widely differing prognosis, together with the possibility that terminology and registration practice varied systematically between regions. Most notably, the North had the highest incidence and survival rates for the subgroup of fibrosarcoma and allied tumours, and the possibility that this was attributable to inclusion of some cases of non-malignant conditions such as fibromatosis could not be excluded (Pastore *et al*, 2006b). The subgroup of ‘Other specified soft tissue sarcomas’, for which the North and South regions had markedly higher survival rates than the BI and West, is also very heterogeneous and includes tumour types with widely differing survival. The difference in survival between the BI and the North was twice as large for all soft tissue sarcomas combined, as it was for rhabdomyosarcoma, the most well-defined and least heterogeneous subgroup in European children. The differences between the BI and South (which had the highest survival of any European region for rhabdomyosarcoma) were similar for rhabdomyosarcoma and for all soft tissue sarcomas. In both ACCIS and EUROCARE 3, interregional variation in survival from STS diminished between the 1970s and 1990s (Gatta *et al*, 2005; Coebergh *et al*, 2006; Pastore *et al*, 2006b; Stiller *et al*, 2006b).

For all leukaemias combined, there was no significant difference between any of the four non-East regions of Europe. However, observed survival for ALL was statistically better in the North and West regions than in BI and South (Coebergh *et al*, 2006). There was no significant difference in outcome for acute non-lymphoblastic leukaemia between the same regions. As organisation of specialist care for children with leukaemia in the UK is similar regardless of subtype, this suggests that differences in treatment rather than other aspects of care underlie the survival difference. Indeed, as treatment protocols for ALL were changed in the late 1990s to introduce more sustained intensification blocks, overall survival has continued to increase, with 3-year overall survival having reached over 90% in the most recent quinquennium (Vora *et al*, 2006; Stiller, 2007).

For children with osteosarcoma, survival was lower in the BI than in the North, West and South (Stiller *et al*, 2006b). There was no such interregional difference in observed survival for Ewing's sarcoma. A contributing factor may have been different approaches to treatment of osteosarcoma during the period studied by ACCIS. Over the entire period covered by the ACCIS survival comparisons (1988–1997), the standard chemotherapy for osteosarcoma in the BI was the two drug combination of cisplatin and doxorubicin, whereas the majority of the other European sarcoma study groups were using multidrug combinations including high dose methotrexate (Souhami *et al*, 1997; Fuchs *et al*, 1998). Although the European Osteosarcoma Intergroup (EOI) randomised studies did not show significant benefit for any of the multidrug combinations tested against the two drug combination, their EFS was at the lower end of the international range. A three drug combination has now been accepted as the standard for the current joint European–American osteosarcoma trial in which the EOI participates (EURAMOS 1; www.euramos.org).

The category of central nervous system tumours presents the most challenges for data comparisons, due to national variation in coding, registration of non-malignant tumours and the low proportion of tumours with a microscopically verified diagnosis. For the category ‘other gliomas’, 58% were diagnosed only clinically with large interregional variation (Peris-Bonet *et al*, 2006). There was a high level of interregional variation in the relative frequencies of cases in the subgroups IIIa (ependymoma, including choroid plexus tumours), IIIb (astrocytoma) and IIId (other glioma), resulting at least in part from differences in diagnostic and classification criteria. Most notably, the ratio of age-standardised incidence rates for astrocytoma to other glioma, which was 3.8 : 1 overall, ranged for individual regions from 1.2 : 1 in the North (influenced by there being no separate code for ‘astrocytoma’ in Finland) to 5.5 : 1 in the East. As discussed earlier, this heterogeneity does not permit meaningful comparisons. In an attempt to overcome this problem, survival rates were analysed for a category of ‘glioma-related’ tumours, which combined these three subgroups. Even for this combined category, however, there was considerable interregional variation in recorded incidence. The North had an especially high overall incidence rate of 26.0 per million, probably reflecting higher rates of diagnosis and registration for low-grade tumours, whereas incidence rates were lower in the West when compared with the BI as the reference region. These differences may explain the better survival of all brain tumours in the North compared with the other three regions, BI, South and West.

Survival within the diagnostic subgroups varied according to the geographical region of residence. Children with PNET had lower survival in the BI or East than in the other three regions. Classification and coding of PNET may be assumed to be fairly consistent internationally. Therefore, it is likely that at least some of this survival difference is due to treatment approaches. For example, a substantial proportion of children with non-metastatic medulloblastoma were treated with radiotherapy alone during the 1990s, in contrast to the greater use of adjuvant chemotherapy in other European countries (Taylor *et al*, 2003). Children with brain tumours have lagged behind other groups of childhood cancers in accessing multidisciplinary specialised care. During the ACCIS study period, only two-thirds of children diagnosed with a brain tumour in Great Britain were referred to a UKCCSG centre (Table 1). This has subsequently increased to 85% in the period 1996–2000 and has coincided with a continued improvement in survival (Figure 3).

## DISCUSSION AND CONCLUSIONS

We have described the potential confounding factors in performing survival comparisons across Europe. We have taken the example of the BI comparisons to explore possible reasons for such differences. The reported survival differences are small in absolute terms and often not statistically significant for individual tumour types. However, the consistency of the findings regarding the relative ranking of the BI in comparison with the Nordic countries and the West, contributed mainly by the former West Germany in both the Eurocare and ACCIS analyses, requires further examination. It is incumbent on clinical investigators to take note of trends and identify areas for improvement. If we accept that survival levels seen in the Nordic countries represent those that the BI could reasonably aspire to at the current time, we need to consider the factors that could theoretically lead to an improvement in survival. Access to specialist care for children with cancer has been well established through the Children's Cancer and Leukaemia Group (CCLG, formerly UK Children's Cancer Study Group (UKCCSG) since the mid-1980s. By the early 2000s, 90% of children with the major childhood cancers were being referred to CCLG centres (Table 1).

The data on neuroblastoma suggest that for some diagnostic categories, it is possible that children are diagnosed at a later stage than their northern European or German counterparts. This may reflect differences in patterns of primary care and child health checks for the young age group in which embryonal tumours typically present. The National Institute for Health and Clinical Excellence (NICE) referral guidelines for suspected cancer, published in June 2005 and applicable in England and Wales, include specific guidance on when to suspect cancer in children (NICE, 2005a). These should improve urgent referral to secondary paediatric services for more timely investigation.

Treatment must be considered as a possible contributing factor to the observed survival differences. Referral to a specialist centre and treatment within a clinical trial are generally viewed as ‘best practice’ for all childhood cancers and there is evidence that treatment within a clinical trial is associated with better survival (Stiller and Eatock, 1999). This approach is endorsed in the recently published NICE guidance document: ‘Improving Outcomes in Children and Young People with Cancer’ (NICE, 2005b). This document also emphasises the importance of multidisciplinary team working to implement the complex diagnostic and therapeutic requirements for effective cancer treatment in this age group.

**Figure 2:**
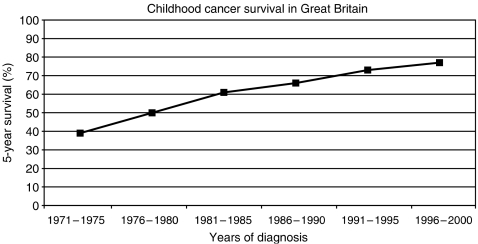
Actuarial 5-year cumulative survival for all childhood cancers diagnosed in Great Britain (England, Wales, Scotland) during the period 1971–2000. Source: Stiller C (2007) *Childhood Cancer in Britain: Incidence, Survival and Mortality*, Oxford University Press: Oxford, UK.

**Figure 3:**
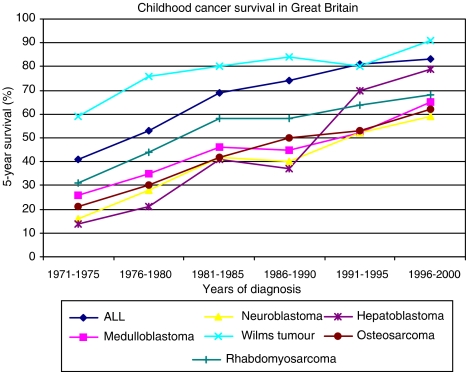
Actuarial 5-year cumulative survival for specified childhood cancers diagnosed in Great Britain during the period 1971–2000. Source: Stiller C (2007) *Childhood Cancer in Britain: Incidence, Survival and Mortality*, Oxford University Press: Oxford, UK.

In the period covered by the ACCIS comparisons (1988–1997), 81% of all children with cancer in the BI were referred to a CCLG (UKCCSG) centre. The majority were enrolled in a relevant national clinical trial. Central nervous system tumours were the main category where the proportion of children referred for specialist oncology care was low and for which there were very few open clinical trials. By the early 2000s, the referral rate had improved to 85% and there is now a comprehensive portfolio of clinical trials for childhood brain tumours. Since the early 1990s, there has been an increasing trend for the CCLG (UKCCSG) to participate in international collaborative clinical trials, initially in solid tumours and more recently in leukaemias. The success of international collaboration in clinical trials is exemplified by the dramatic improvement in survival for hepatoblastoma from 37% in the era before the opening of the first SIOPEL trial in 1990 to 70% in the next quinquennium (Figure 3). The improvement in survival for all cancers and for the tumour subgroups where the BI differed from the best regions in Europe continues into the most recent period for which mature 5-year survival data are available and shows no signs of levelling off (Figures 2 and 3). In England and Wales, implementation of the NICE guidance means that multidisciplinary teams have been strengthened in many centres. Similar guidance is being implemented in Scotland. These changes in service delivery and increasing international collaboration are expected to have a continued positive effect on outcome. It is therefore essential that systems remain in place to permit ongoing geographical comparisons of incidence of and survival from childhood cancer. Clinical investigators and cancer registries should also pursue ways to collect more detailed information to permit analysis of hypotheses as to why overall survival rates continue to increase even during time periods when the same clinical trial protocols are running. Such information should include disease free not just overall survival together with participation rates in randomised clinical trials. Such analyses provide an important basis for countries to examine the impact of their national strategies to develop services for children with cancer.

## Figures and Tables

**Figure 1:**
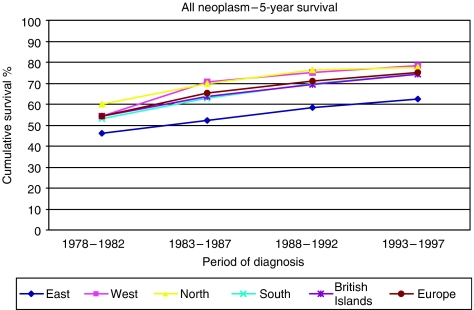
Five-year actuarial cumulative survival for all childhood cancers, by European region and period of diagnosis (source: Magnani *et al*, 2006) Trends in survival after childhood cancer in Europe, 1978–1997: the ACCIS project. *Eur J Cancer* **42**:1981–2005). For details of the countries included in each of the regions, see main text. Reproduced with the permission of the *European Journal of Cancer*.

**Table 1:** Percentage of children with cancer in Great Britain initially referred to a CCLG (formerly UKCCSG) centre by ICCC-3 main diagnostic group and for all groups combined

**Diagnostic groups**	**1988–1995**	**1996–2000**	**2001–2002**
I Leukaemia	87	93	95
II Lymphoma	86	91	94
III CNS	65	85	85
IV Sympathetic Nervous system	96	98	98
V Retinoblastoma	88	93	91
VI Renal tumours	94	98	99
VII Hepatic tumours	88	90	97
VIII Bone tumours	74	94	92
IX Soft tissue sarcoma	85	90	85
X Germ cell and gonadal	77	82	90
XI Melanoma and other carcinoma	23	36	35
XII Other	11	35	36
All cancers combined (I–XII)	79	88	90

Results are given for the period corresponding to the ACCIS analyses (data for England and Wales in the geographical comparison of survival was cutoff at 1995, (Sankila *et al*, 2006), the more recent quinquennium and the most recent period (2001–2002) for which registration data are virtually complete. Source: National Registry of Childhood Tumours, Oxford.
